# 6,11-Dihydro­dibenz[*b*,*e*]oxepin-11-one

**DOI:** 10.1107/S1600536809053409

**Published:** 2009-12-16

**Authors:** Jerry P. Jasinski, Q. N. M. Hakim Al-arique, Ray J. Butcher, H. S. Yathirajan, B. Narayana

**Affiliations:** a Department of Chemistry, Keene State College, 229 Main Street, Keene, NH 03435-2001, USA; b Department of Studies in Chemistry, University of Mysore, Manasagangotri, Mysore 570 006, India; c Department of Chemistry, Howard University, 525 College Street NW, Washington DC 20059, USA; d Department of Studies in Chemistry, Mangalore University, Mangalagangotri 574 199, India

## Abstract

In the title compound, C_14_H_10_O_2_, the seven-membered oxepine ring adopts a twist-boat conformation with a dihedral angle between the mean planes of the two fused benzene rings of 42.0 (1)°. In the crystal, mol­ecules are linked into chains propagating along the *c* axis by inter­molecular C—H⋯O hydrogen bonds and the chains are arranged in layers parallel to (100).

## Related literature

The dibenz[*b*,*e*]oxepin nucleus constitutes the fundamental structure of many products with biological activity, see: Kumazawa *et al.* (1994 ▶). For dibenzo[*c*,*e*]thiepine derivatives and their chiroptical properties, see: Truce *et al.* (1956 ▶); Tomascovic *et al.* (2000 ▶). For comparative NMR and IR spectral, X-ray structural and theoretical studies of eight related 6-aryl­idenedibenzo[*b*,*e*]thiepin-11-one-5,5-dioxides, see: Kolehmainen *et al.* (2007 ▶). For related structures, see: Bandoli & Nicolini (1982 ▶); Blaton *et al.* (1995 ▶); Ieawsuwan *et al.* (2006 ▶); Linden *et al.* (2004 ▶); Roszak *et al.* (1996 ▶); Yoshinari & Konno (2009 ▶); Zhang *et al.* (2008 ▶,2008*a* ▶). For DFT calculations, see: Hehre *et al.* (1986 ▶); Schmidt & Polik (2007 ▶). For the *GAUSSIAN03* program package, see: Frisch *et al.* (2004 ▶). For a description of the Cambridge Structural Database, see: Allen (2002 ▶).
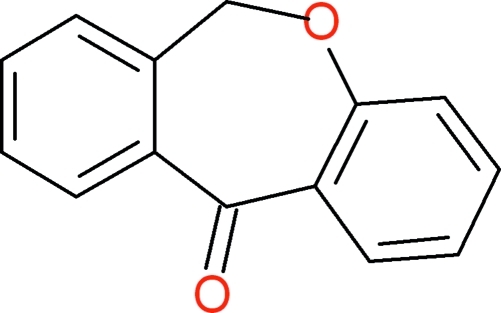


## Experimental

### 

#### Crystal data

C_14_H_10_O_2_
*M* *_r_* = 210.22
Monoclinic, 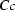

*a* = 16.5065 (18) Å
*b* = 4.0806 (7) Å
*c* = 15.0392 (17) Å
β = 93.654 (10)°
*V* = 1010.9 (2) Å^3^
*Z* = 4
Mo *K*α radiation
μ = 0.09 mm^−1^
*T* = 110 K
0.53 × 0.27 × 0.23 mm

#### Data collection

Oxford Diffraction Gemini R CCD diffractometer
Absorption correction: multi-scan (*CrysAlis RED*; Oxford Diffraction, 2007 ▶) *T*_min_ = 0.864, *T*_max_ = 1.000
1472 measured reflections
968 independent reflections
953 reflections with *I* > 2σ(*I*)
*R*_int_ = 0.019

#### Refinement

*R*[*F* ^2^ > 2σ(*F* ^2^)] = 0.040
*wR*(*F* ^2^) = 0.108
*S* = 1.06
968 reflections
145 parameters
2 restraints
H-atom parameters constrained
Δρ_max_ = 0.24 e Å^−3^
Δρ_min_ = −0.24 e Å^−3^

### 

Data collection: *CrysAlis PRO* (Oxford Diffraction, 2007 ▶); cell refinement: *CrysAlis PRO*; data reduction: *CrysAlis PRO*; program(s) used to solve structure: *SHELXS97* (Sheldrick, 2008 ▶); program(s) used to refine structure: *SHELXL97* (Sheldrick, 2008 ▶); molecular graphics: *SHELXTL* (Sheldrick, 2008 ▶); software used to prepare material for publication: *SHELXTL*.

## Supplementary Material

Crystal structure: contains datablocks global, I. DOI: 10.1107/S1600536809053409/ci2982sup1.cif

Structure factors: contains datablocks I. DOI: 10.1107/S1600536809053409/ci2982Isup2.hkl

Additional supplementary materials:  crystallographic information; 3D view; checkCIF report

## Figures and Tables

**Table 1:** Hydrogen-bond geometry (Å, °)

*D*—H⋯*A*	*D*—H	H⋯*A*	*D*⋯*A*	*D*—H⋯*A*
C10—H10*A*⋯O1^i^	0.95	2.57	3.380 (3)	143

Symmetry code: (i) 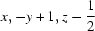.

**Fig. 1.**
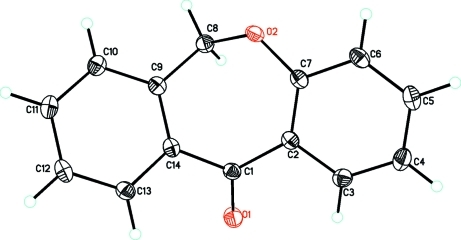
Molecular structure of the title compound, showing atom-labeling scheme and 50% probability displacement ellipsoids.

**Fig. 2.**
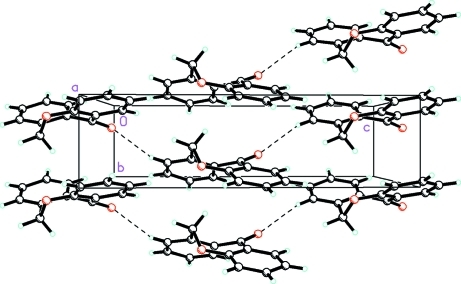
Packing diagram of the title compound, viewed down the a axis. Dashed lines indicate weak intermolecular C—H···O hydrogen bonds.

## supplementary crystallographic information

### Comment

The title compound is used as an intermediate for the synthesis of doxepin, which is a psychotropic agent with tricyclic antidepressant and anxiolytic properties. Doxepin is a tricyclic compound and displays a range of pharmacological actions including maintaining adrenergic innervation. Its mechanism of action is not fully understood, but it appears to block re-uptake of monoaminergic neurotransmitters into presynaptic terminals. It also possesses anticholinergic activity and modulates antagonism of histamine H(1)- and H(2)-receptors. The dibenz[b,e]oxepin nucleus constitutes the fundamental structure of many products with biological activity (Kumazawa *et al.*, 1994). The dibenzo[c,e]thiepine derivatives (Truce *et al.*, 1956) exhibit remarkable chiroptical properties (Tomascovic *et al.*, 2000). The comparative NMR and IR spectral, X-ray structural and theoretical studies of eight related 6-arylidenedibenzo[b,e]thiepin-11-one-5,5-dioxides have been reported (Kolehmainen *et al.*, 2007). In view of the importance of oxepines, this paper reports the crystal structure of the title compound.

The seven-membered oxepin ring adopts a twist-boat conformation with the dihedral angle between the mean planes of the two fused benzene rings measuring 42.0 (1)° (Fig. 1). This conformation is assisted by *sp*^3^ hybridization of atoms C8 and O2 within the ring. The ketone oxygen atom (O1) lies in an equatorial position from the ring on opposite sides of the C8 and O2 atoms (C3—C2—C1—O1 = -19.1 (4)° and C13—C14—C1—O1 = 35.3 (4)°). Bond lengths and angles are all within expected ranges (Allen, 2002). The molecules are linked into chains propagating along the *c*-axis by C10–H10A···O1 intermolecular hydrogen bonds and the chains are arranged in layers parallel to the (100) (Fig. 2).

After a density functional theory (DFT) computational calculation at the 6–31-G(*d*) level (Hehre *et al.*, 1986; Schmidt & Polik, 2007) with the GAUSSIAN03 program package (Frisch *et al.*, 2004), the angle between the mean planes of the two benzene rings becomes 34.8 (2)°, a decrease of 7.2 (2)°. The C3—C2—C1—O1 and C13—C14—C1—O1 torsion angles become -8.7 (5)°) and 26.1 (5)°, a decrease of 10.3 (5)° and 9.1 (8)°, respectively. This suggests that the crystal packing is influenced by the weak C—H···O intermolecular hydrogen bonding interactions.

### Experimental

The title compound was obtained as a gift sample from *R. L*. Fine Chem, Bangalore, India. The compound was used without further purification. X-ray quality crystals (m.p. 327–329 K) were obtained by slow evaporation from a methanol solution.

### Refinement

All of the H atoms were placed in their calculated positions and then refined using the riding model with C-H = 0.95–0.99 Å, and with *U*_iso_(H) = 1.18–1.21*U*_eq_(C).

### Crystal data

**Table:**

C_14_H_10_O_2_	*F*(000) = 440
*M**_r_* = 210.22	*D*_x_ = 1.381 Mg m^−^^3^
Monoclinic, *C**c*	Mo *K*α radiation, λ = 0.71073 Å
Hall symbol: C -2yc	Cell parameters from 1157 reflections
*a* = 16.5065 (18) Å	θ = 6.0–73.7°
*b* = 4.0806 (7) Å	µ = 0.09 mm^−^^1^
*c* = 15.0392 (17) Å	*T* = 110 K
β = 93.654 (10)°	Thick needle, colorless
*V* = 1010.9 (2) Å^3^	0.53 × 0.27 × 0.23 mm
*Z* = 4	

### Data collection

**Table:**

Oxford Diffraction Gemini R CCD diffractometer	968 independent reflections
Radiation source: fine-focus sealed tube	953 reflections with *I* > 2σ(*I*)
graphite	*R*_int_ = 0.019
Detector resolution: 10.5081 pixels mm^-1^	θ_max_ = 26.3°, θ_min_ = 3.8°
φ and ω scans	*h* = −15→20
Absorption correction: multi-scan (*CrysAlis RED*; Oxford Diffraction, 2007)	*k* = −4→2
*T*_min_ = 0.864, *T*_max_ = 1.000	*l* = −18→17
1472 measured reflections	

### Refinement

**Table:**

Refinement on *F*^2^	Primary atom site location: structure-invariant direct methods
Least-squares matrix: full	Secondary atom site location: difference Fourier map
*R*[*F*^2^ > 2σ(*F*^2^)] = 0.040	Hydrogen site location: inferred from neighbouring sites
*wR*(*F*^2^) = 0.108	H-atom parameters constrained
*S* = 1.06	*w* = 1/[σ^2^(*F*_o_^2^) + (0.0835*P*)^2^ + 0.4174*P*] where *P* = (*F*_o_^2^ + 2*F*_c_^2^)/3
968 reflections	(Δ/σ)_max_ = 0.001
145 parameters	Δρ_max_ = 0.24 e Å^−^^3^
2 restraints	Δρ_min_ = −0.24 e Å^−^^3^

### Special details

**Table:**

Geometry. All e.s.d.'s (except the e.s.d. in the dihedral angle between two l.s. planes) are estimated using the full covariance matrix. The cell e.s.d.'s are taken into account individually in the estimation of e.s.d.'s in distances, angles and torsion angles; correlations between e.s.d.'s in cell parameters are only used when they are defined by crystal symmetry. An approximate (isotropic) treatment of cell e.s.d.'s is used for estimating e.s.d.'s involving l.s. planes.
Refinement. Refinement of *F*^2^ against ALL reflections. The weighted *R*-factor *wR* and goodness of fit *S* are based on *F*^2^, conventional *R*-factors *R* are based on *F*, with *F* set to zero for negative *F*^2^. The threshold expression of *F*^2^ > σ(*F*^2^) is used only for calculating *R*-factors(gt) *etc*. and is not relevant to the choice of reflections for refinement. *R*-factors based on *F*^2^ are statistically about twice as large as those based on *F*, and *R*- factors based on ALL data will be even larger.

### Fractional atomic coordinates and isotropic or equivalent isotropic displacement parameters (Å^2^)

**Table:**

	*x*	*y*	*z*	*U*_iso_*/*U*_eq_	
O1	0.45613 (12)	0.6693 (6)	0.53696 (13)	0.0390 (6)	
O2	0.63969 (10)	0.7966 (4)	0.34730 (11)	0.0255 (4)	
C1	0.49884 (15)	0.7517 (7)	0.47743 (17)	0.0252 (5)	
C2	0.58727 (16)	0.8141 (6)	0.49769 (17)	0.0239 (5)	
C3	0.61004 (16)	0.8726 (7)	0.58847 (17)	0.0293 (6)	
H3A	0.5707	0.8400	0.6311	0.035*	
C4	0.68631 (18)	0.9743 (8)	0.61780 (19)	0.0343 (7)	
H4A	0.6997	1.0074	0.6795	0.041*	
C5	0.74432 (17)	1.0287 (7)	0.5551 (2)	0.0333 (6)	
H5A	0.7970	1.1056	0.5740	0.040*	
C6	0.72459 (16)	0.9703 (7)	0.46615 (19)	0.0285 (6)	
H6A	0.7640	1.0079	0.4240	0.034*	
C7	0.64779 (15)	0.8571 (6)	0.43686 (17)	0.0236 (5)	
C8	0.57772 (16)	0.5685 (7)	0.31616 (18)	0.0275 (6)	
H8A	0.5774	0.3803	0.3577	0.033*	
H8B	0.5906	0.4841	0.2570	0.033*	
C9	0.49518 (16)	0.7233 (6)	0.30917 (17)	0.0236 (5)	
C10	0.45306 (17)	0.7704 (7)	0.22715 (18)	0.0280 (6)	
H10A	0.4768	0.7045	0.1741	0.034*	
C11	0.37673 (18)	0.9126 (8)	0.22225 (18)	0.0312 (6)	
H11A	0.3483	0.9431	0.1659	0.037*	
C12	0.34162 (17)	1.0107 (8)	0.29936 (19)	0.0311 (6)	
H12A	0.2898	1.1129	0.2958	0.037*	
C13	0.38244 (15)	0.9588 (7)	0.38147 (18)	0.0275 (6)	
H13A	0.3579	1.0205	0.4344	0.033*	
C14	0.45922 (15)	0.8167 (6)	0.38669 (17)	0.0227 (5)	

### Atomic displacement parameters (Å^2^)

**Table:**

	*U*^11^	*U*^22^	*U*^33^	*U*^12^	*U*^13^	*U*^23^
O1	0.0282 (10)	0.0601 (15)	0.0289 (10)	−0.0057 (10)	0.0042 (8)	0.0139 (10)
O2	0.0203 (9)	0.0282 (9)	0.0285 (10)	0.0004 (8)	0.0056 (7)	−0.0020 (8)
C1	0.0239 (12)	0.0259 (12)	0.0261 (12)	−0.0002 (10)	0.0039 (10)	0.0027 (10)
C2	0.0239 (12)	0.0197 (12)	0.0280 (13)	0.0021 (9)	0.0012 (9)	0.0032 (9)
C3	0.0316 (14)	0.0302 (14)	0.0261 (12)	0.0035 (11)	0.0027 (10)	0.0044 (11)
C4	0.0386 (15)	0.0351 (16)	0.0282 (14)	0.0019 (12)	−0.0067 (12)	−0.0022 (12)
C5	0.0247 (12)	0.0277 (14)	0.0463 (17)	0.0002 (11)	−0.0073 (11)	−0.0028 (12)
C6	0.0226 (11)	0.0231 (13)	0.0401 (14)	0.0010 (9)	0.0035 (10)	−0.0004 (11)
C7	0.0215 (11)	0.0182 (12)	0.0309 (13)	0.0038 (9)	0.0005 (9)	0.0028 (10)
C8	0.0278 (13)	0.0252 (14)	0.0295 (12)	0.0026 (10)	0.0010 (10)	−0.0054 (10)
C9	0.0240 (11)	0.0181 (12)	0.0288 (13)	−0.0041 (9)	0.0018 (9)	−0.0004 (9)
C10	0.0314 (13)	0.0245 (13)	0.0277 (12)	−0.0052 (10)	−0.0015 (10)	−0.0019 (10)
C11	0.0331 (13)	0.0310 (15)	0.0285 (13)	−0.0042 (11)	−0.0066 (10)	0.0034 (11)
C12	0.0218 (11)	0.0300 (14)	0.0410 (15)	0.0013 (9)	−0.0034 (10)	0.0061 (11)
C13	0.0237 (12)	0.0290 (14)	0.0300 (12)	−0.0013 (10)	0.0042 (9)	0.0015 (10)
C14	0.0215 (10)	0.0178 (13)	0.0286 (13)	−0.0028 (9)	−0.0003 (9)	0.0037 (9)

### Geometric parameters (Å, °)

**Table:**

O1—C1	1.221 (3)	C6—H6A	0.95
O2—C7	1.367 (3)	C8—C9	1.499 (3)
O2—C8	1.439 (3)	C8—H8A	0.99
C1—C2	1.494 (3)	C8—H8B	0.99
C1—C14	1.499 (3)	C9—C10	1.390 (4)
C2—C7	1.408 (3)	C9—C14	1.395 (4)
C2—C3	1.413 (4)	C10—C11	1.385 (4)
C3—C4	1.372 (4)	C10—H10A	0.95
C3—H3A	0.95	C11—C12	1.388 (4)
C4—C5	1.403 (4)	C11—H11A	0.95
C4—H4A	0.95	C12—C13	1.385 (4)
C5—C6	1.378 (4)	C12—H12A	0.95
C5—H5A	0.95	C13—C14	1.391 (4)
C6—C7	1.394 (3)	C13—H13A	0.95
			
C7—O2—C8	117.40 (19)	O2—C8—H8A	109.2
O1—C1—C2	120.0 (2)	C9—C8—H8A	109.2
O1—C1—C14	118.5 (2)	O2—C8—H8B	109.2
C2—C1—C14	121.3 (2)	C9—C8—H8B	109.2
C7—C2—C3	116.8 (2)	H8A—C8—H8B	107.9
C7—C2—C1	127.8 (2)	C10—C9—C14	119.2 (3)
C3—C2—C1	115.0 (2)	C10—C9—C8	121.4 (2)
C4—C3—C2	123.0 (3)	C14—C9—C8	119.3 (2)
C4—C3—H3A	118.5	C11—C10—C9	120.5 (3)
C2—C3—H3A	118.5	C11—C10—H10A	119.8
C3—C4—C5	118.9 (3)	C9—C10—H10A	119.8
C3—C4—H4A	120.5	C10—C11—C12	120.2 (2)
C5—C4—H4A	120.5	C10—C11—H11A	119.9
C6—C5—C4	119.7 (3)	C12—C11—H11A	119.9
C6—C5—H5A	120.1	C13—C12—C11	119.7 (3)
C4—C5—H5A	120.1	C13—C12—H12A	120.1
C5—C6—C7	121.2 (3)	C11—C12—H12A	120.1
C5—C6—H6A	119.4	C12—C13—C14	120.2 (2)
C7—C6—H6A	119.4	C12—C13—H13A	119.9
O2—C7—C6	113.6 (2)	C14—C13—H13A	119.9
O2—C7—C2	126.1 (2)	C9—C14—C13	120.1 (2)
C6—C7—C2	120.3 (2)	C9—C14—C1	121.9 (2)
O2—C8—C9	112.0 (2)	C13—C14—C1	117.9 (2)
			
O1—C1—C2—C7	168.6 (3)	O2—C8—C9—C10	113.0 (3)
C14—C1—C2—C7	−16.9 (4)	O2—C8—C9—C14	−68.2 (3)
O1—C1—C2—C3	−19.1 (4)	C14—C9—C10—C11	0.9 (4)
C14—C1—C2—C3	155.4 (2)	C8—C9—C10—C11	179.7 (3)
C7—C2—C3—C4	1.6 (4)	C9—C10—C11—C12	0.2 (4)
C1—C2—C3—C4	−171.6 (3)	C10—C11—C12—C13	−1.5 (4)
C2—C3—C4—C5	1.1 (5)	C11—C12—C13—C14	1.6 (4)
C3—C4—C5—C6	−1.9 (4)	C10—C9—C14—C13	−0.8 (4)
C4—C5—C6—C7	−0.1 (4)	C8—C9—C14—C13	−179.6 (2)
C8—O2—C7—C6	156.5 (2)	C10—C9—C14—C1	175.7 (2)
C8—O2—C7—C2	−23.3 (3)	C8—C9—C14—C1	−3.1 (4)
C5—C6—C7—O2	−176.9 (2)	C12—C13—C14—C9	−0.5 (4)
C5—C6—C7—C2	2.9 (4)	C12—C13—C14—C1	−177.1 (2)
C3—C2—C7—O2	176.3 (2)	O1—C1—C14—C9	−141.2 (3)
C1—C2—C7—O2	−11.6 (4)	C2—C1—C14—C9	44.2 (4)
C3—C2—C7—C6	−3.5 (4)	O1—C1—C14—C13	35.3 (4)
C1—C2—C7—C6	168.6 (3)	C2—C1—C14—C13	−139.2 (3)
C7—O2—C8—C9	79.7 (3)		

### Hydrogen-bond geometry (Å, °)

**Table:**

*D*—H···*A*	*D*—H	H···*A*	*D*···*A*	*D*—H···*A*
C10—H10A···O1^i^	0.95	2.57	3.380 (3)	143

Symmetry codes: (i) *x*, −*y*+1, *z*−1/2.
